# Roles of human papillomavirus in cancers: oncogenic mechanisms and clinical use

**DOI:** 10.1038/s41392-024-02083-w

**Published:** 2025-01-24

**Authors:** Yu Zhang, Ke Qiu, Jianjun Ren, Yu Zhao, Ping Cheng

**Affiliations:** 1 Department of Biotherapy, Cancer Center and State Key Laboratory of Biotherapy, West China Hospital, Sichuan University, Chengdu, Sichuan 610041 China; 2 Department of Otolaryngology-Head & Neck Surgery, West China Hospital, Sichuan University, Chengdu, Sichuan 610041 China

**Keywords:** Oncogenes, Gynaecological cancer, Head and neck cancer

## Abstract

Human papillomaviruses, particularly high-risk human papillomaviruses, have been universally considered to be associated with the oncogenesis and progression of various cancers. The genome of human papillomaviruses is circular, double-stranded DNA that encodes early and late proteins. Each of the proteins is of crucial significance in infecting the epithelium of host cells persistently and supporting viral genome integrating into host cells. Notably, E6 and E7 proteins, classified as oncoproteins, trigger the incidence of cancers by fostering cell proliferation, hindering apoptosis, evading immune surveillance, promoting cell invasion, and disrupting the balance of cellular metabolism. Therefore, targeting human papillomaviruses and decoding molecular mechanisms by which human papillomaviruses drive carcinogenesis are of great necessity to better treat human papillomaviruses-related cancers. Human papillomaviruses have been applied clinically to different facets of human papillomavirus-related cancers, including prevention, screening, diagnosis, treatment, and prognosis. Several types of prophylactic vaccines have been publicly utilized worldwide and have greatly decreased the occurrence of human papillomavirus-related cancers, which have benefited numerous people. Although various therapeutic vaccines have been developed and tested clinically, none of them have been officially approved to date. Enhancing the efficacy of vaccines and searching for innovative technologies targeting human papillomaviruses remain critical challenges that warrant continuous research and attention in the future.

## Introduction

Human papillomaviruses (HPVs), recognized as one of the most common sexually transmitted viruses, can infect skin and mucosa.^1,2^ HPVs can be sorted into five genera: Alpha, Beta, Gamma, Mu, and Nu.^3^ HPVs from the Beta and Gamma genera can elicit inapparent infections of the skin. Alpha-HPVs are associated with various clinical conditions ranging from benign warts to cancers. For instance, HPV-6 and HPV-11 are connected with oral focal hyperplasia and anogenital warts. HPV-3, HPV-10, HPV-28 are closely linked with plane warts, whereas HPV-2, HPV-7, and HPV-40 are associated with common warts. Among Alpha-HPVs, only a small part of Alpha-HPVs is oncogenic, which is termed as high-risk HPVs (hr-HPVs) (Table 1). Most HPV infections are transient and asymptomatic without causing any health problems. Persistent infection with hr-HPVs can cause precancerous lesions, thus evolving into various cancers, including head and neck squamous cell carcinoma (HNSCC), cervical cancer (CC), anal cancer, as well as vulvar, penile, and vaginal cancers that are less prevalent.

**Table 1:** hr-HPVs and associated diseases

Genera	Types	Diseases
α-5	51, 82	Anogenital cancers
α-6	56	Anogenital cancers
α-7	18, 39, 45, 59, 68	Anogenital cancers, oropharyngeal carcinoma
α-9	16, 31, 33, 35, 52, 58	Anogenital cancers, oropharyngeal carcinoma
α-11	73	Anogenital cancers

In 2020, the World Health Organization (WHO) revised the classification of CC into two subtypes: HPV-associated and HPV-independent. In addition, HPV-associated adenocarcinoma has a better prognosis than HPV-independent adenocarcinoma in CC.^4^ Besides, the American Joint Committee on Cancer has set a specific staging system for HPV-positive oropharyngeal squamous cell carcinoma (OPSCC), which is a specific type of HNSCC. Research has confirmed that HPV-positive OPSCC is more sensitive to radiotherapy and chemotherapy, with a less aggressive phenotype and better prognosis.^5,6^ Moreover, HPV infection not only provides reliable biomarkers for the screening and early diagnosis of hr-HPV-related cancers, but also offers a series of potential therapeutic targets for treating hr-HPV-related cancers. Various vaccines targeting hr-HPVs have been developed to prevent and treat hr-HPV-related cancers, which will be narrated in detail in the following text.

**Fig. 1:**
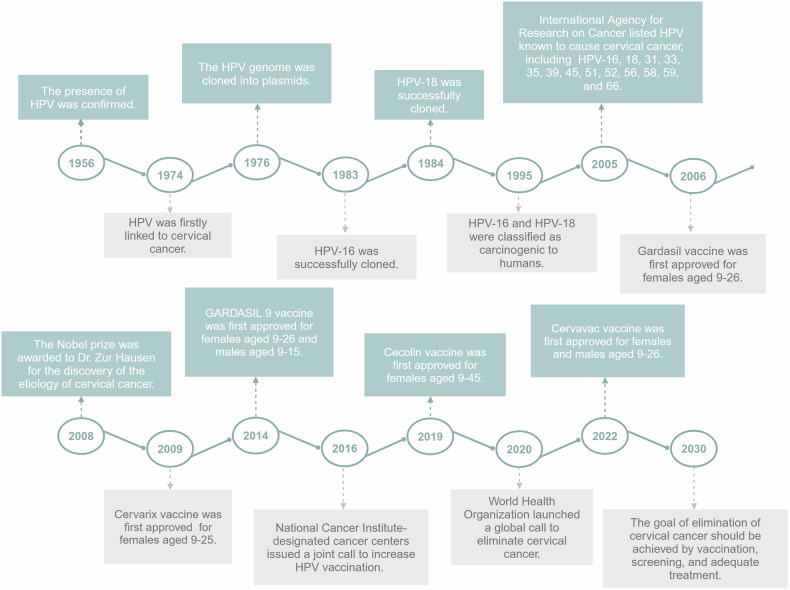
A timeline of HPV research. Since the presence was identified in 1956, study on HPV has continued to date. Apart from oncogenic mechanisms, vaccines targeting HPV have been developed. Moreover, WHO has launched strategies for worldwide CC elimination. This figure was created with BioRender (www.bioender.com)

The study of HPVs is time-honored (Fig. 1). In 1956, with the aid of the electron microscopic technique, the presence of HPV was identified and the feature of HPV was confirmed. Harald zur Hausen firstly proposed a hypothesis that HPV may be related with CC in 1974. The HPV genome was cloned into plasmids in 1976, providing methods for studying the underlying molecular mechanisms of HPV. And more and more research found the existence of HPV in lesions of cervix.^7,8^ Research on HPV saw significant advancement in the 1970s and 1980s, in which HPV-16 and HPV-18 were successfully cloned in 1983 and 1984, respectively.^9^ Moreover, HPV-16 and HPV-18 were classified as oncogenic in 1995. The characteristic of viral DNA such as immortalization was found and the oncogenic mechanisms of E6 and E7 proteins were further studied.^10,11^ Next, significant progress was witnessed in the 1990s and 2000s. Gardasil, Cervarix, and Gardasil-9 were developed and approved to prevent the occurrence of hr-HPV-related cancers. And the WHO has launched a worldwide strategy to eliminate CC in 2030. Nowadays, studies related to HPV include oncogenic signaling mechanisms, the development of various preventative and therapeutic vaccines, and different therapies targeting hr-HPV.

**Fig. 2:**
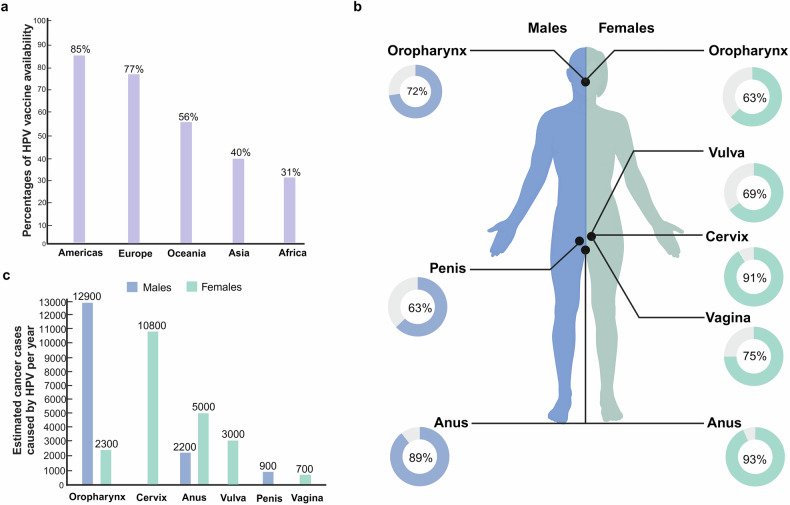
Global HPV vaccine availability and the association between hr-HPVs and cancers. **a** Percentage of HPV vaccine availability among different regions. **b** The association between hr-HPVs and cancers. Cervical cancer, oropharyngeal squamous cell carcinoma, anal cancer, vaginal cancer, vulvar cancer, and penile cancer are well recognized as closely associated with hr-HPVs. **c** Number of cancer cases caused by hr-HPV per year. This figure was created with BioRender (www.bioender.com)

Despite the availability of screening methods and various vaccines, the burden of hr-HPV-related malignancies still exists, which mainly detrimentally affects low- and meddle-income countries (LMICs) due to limited access to prevention and treatment. According to statistics, CC remains the most generally diagnosed cancers in women in 25 countries among 185 countries and CC is the main cause of cancer death in 37 countries especially in LMICs. Moreover, the incidence and mortality of CC ranks in top five in LMICs.^12^ In 2018, LMICs contribute to almost 90% of the mortality of CC. About 31% of LMICs have implemented HPV vaccination compared with 85% of high-income countries. The availability of HPV vaccine varies significantly among different regions (Fig. 2a).^13^ In addition, only 20% of women in LMICs have access to HPV screening, whereas more than 60% of women experience HPV screening in high-income countries.^14,15^

The implications of this review extend beyond understanding the life cycle and oncogenic signaling pathways of hr-HPV infections. It also underscores the role of hr-HPV as a significant biomarker for cancer screening and early detection, as well as a therapeutic target for cancer treatment. As research progresses, these potential targets and biomarkers present opportunities to reduce the global burden of HPV-associated diseases through early intervention, personalized treatment strategies, and potentially curative therapies.

## The epidemiology and current clinical management of hr-HPV-related cancers

It has been widely recognized that persistent hr-HPV infection contributes to certain types of cancers (Fig. 2b) and the number of cancer cases caused by hr-HPV has been estimated according to the Centers for Disease Control and Prevention (CDC) (Fig. 2c).^16,17^ Until now, 15 types of hr-HPVs have been found to be linked with the occurrence and development of CC, among which HPV-16 and HPV-18 contribute to almost 75% of CCs,^18–20^ while HPV-31, 33, 45, 52, and 58 contribute to the other 15–20% of CCs.^21^ CC, known as the fourth most common cancer in women around the world, had almost 604,000 cases and 342,000 deaths in 2020 according to the WHO.^22^ Due to the widespread application of screening and prevention, the incidence of CC has declined dramatically, and there will be an estimated 13,820 new cases in the United States in 2024.^23^ Meanwhile, compared with high-income countries, LMICs in Africa and southern Asia account for a large proportion of hr-HPV-related CCs due to the expensive costs of HPV screening and vaccination.^24^ Besides, a total of 891,453 cases of HNSCCs were estimated worldwide in 2022^12^ and the incidence of HNSCCs is predicted to exceed that of CC in the United States and the UK. It’s also noteworthy that OPSCC has been found to be closely associated with hr-HPVs, with almost 70% of OPSCCs are caused by hr-HPVs, especially HPV-16.^25^ The incidence of hr-HPV-related OPSCC has increased dramatically over the past twenty years in the white population from high-income countries, especially in those with little or no tobacco exposure.^26,27^ Moreover, ~90% of anal cancers are also caused by persistent hr-HPV infection.^23^

ThinPrep cytologic test, HPV test, together with colposcopy has become valuable methods to detect pathological changes of the cervix, which has greatly contributed to the timely intervention of precancerous lesions and has benefited a large number of the female population. Radical hysterectomy with bilateral pelvic lymph node dissection is the standard treatment for early-stage CCs, whilst concurrent chemoradiation, based on drugs containing platinum is the first choice of advanced-stage CC. Moreover, pembrolizumab plus chemotherapy with or without bevacizumab is recommended as the first-line therapy for recurrent or metastatic CC.^4^

Notably, although HPV status has been included as an important staging factor in the 8th edition of the American Joint Committee on Cancer staging system for OPSCC, there are almost no differences in the treatment between hr-HPV-related OPSCC and hr-HPV-unrelated OPSCC.^28^ For early-stage OPSCC, surgery by a double transoral and transcervical approach or intensity-modulated radiotherapy is recommended, whilst cisplatin-based concurrent chemoradiation is the first-line therapy for advanced OPSCC. Meanwhile, nodal condition, tumor diameter, positive surgical margins, degree of differentiation and invasion pattern are also important factors to be considered in the treatment selection and prognostic prediction for OPSCC.^6^

## Life cycle of hr-HPV during infection

HPV is a non-enveloped virus that owns an icosahedral capsid composed of 72 capsomers: 360 copies of the 55 kDa L1 protein and 12 copies of the 74 kDa L2 protein.^29,30^ The HPV genome is a circular, double-stranded DNA about 7–8 kb in size. The HPV genome has three characterized regions: the upstream regulatory region (URR) which is also called the long control region (LCR); the early (E) region; and the late (L) region. Owing to possessing binding sites for E1 and E2 proteins, and transcription elements such as papillomavirus enhancer factor-1, activating protein 1, octamer binding factor-1, transcription enhancer factor-1 (TEF-1) and TEF-2, transcription specificity protein 1, and nuclear factor-1,^31–33^ the URR is responsible for initiating the replication and transcription of HPV DNA. Besides, the URR contains the p97 promoter together with regulatory motifs and is recognized as the region that possesses the highest variation. According to this feature, analysis of URR can be applied to the classification of HPVs.^29,34^ The early region encodes E1, E2, E1^E4, E5, E6, E7, and E8^E2 proteins, which are closely associated with infection, viral replication, and oncogenesis.^29^ The late region encodes L1 and L2 proteins. In addition, the HPV genome contains early (P_E_) and late (P_L_) promoters, as well as early (pA_E_) and late (pA_L_) polyadenylation sites.

**Fig. 3:**
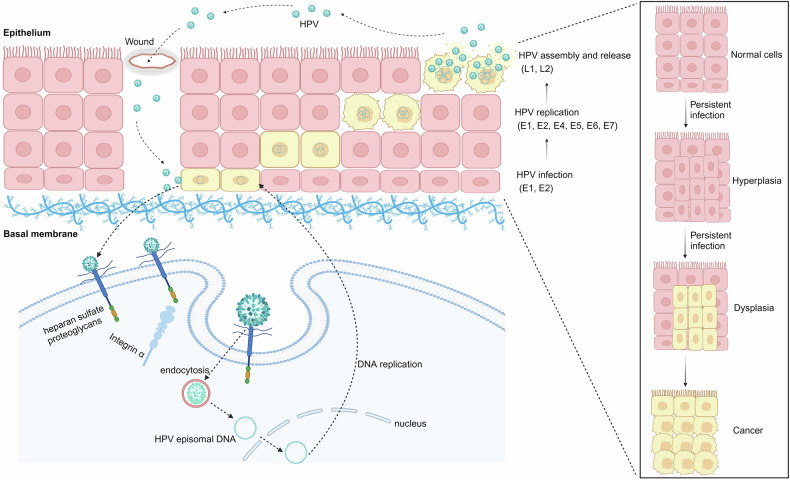
The life cycle of hr-HPVs. HPVs enter the basal layer of the epithelium and undergo different stages: infection, replication, capsid synthesis, virion assembly, and virion release. Early proteins are of great significance during HPV infection. Persistent infection results in hyperplasia and dysplasia. Without intervention, dysplasia has a great possibility to evolve into cancers. This figure was created with BioRender (www.bioender.com)

HPV particles enter the basal layer of the epithelium via minuscule abrasion or wound of epidermis. Then, HPV particles attach to heparan sulfate proteoglycans and secondary receptors such as α_6_-Integrin, which facilitate HPV particles entering into host cells. HPV infection undergoes four stages once entering the basal cells: initial, maintenance, vegetative amplification, as well as virus assembly and release (Fig. 3).

Through endocytosis, HPV particles have access to basal cells. Then, the L1 protein separates from the L2 protein. The L2 protein forms a complex with the HPV genome in the vesicle form, transports to the trans-Golgi and resides until HPV particles enter the nucleus when the nuclear envelope breaks down during mitosis. The promyelocytic leukemia (PML) protein is recruited to the surrounding viral genome prior to the genome entering the nucleus during early interphase. The L2 protein, together with the viral genome, remains at PML-nuclear bodies even in late interphase, which offers a protective environment and a possible position for the initial infection and low-copy number of viral genomes.^35^ Upon getting into the nucleus, the E1 and E2 proteins are expressed and cooperatively connect to the replication origin to initiate DNA replication. HPVs infect the proliferating basal cells of the epithelium to construct the maintenance stage, during which HPV genomes exist in the form of extrachromosomal plasmids^36^ and replicate along with the host genome in the S-phase. Notably, hr-HPVs have the ability to drive cell proliferation in the basal and parabasal layers, distinguishing them from other types of HPVs. DNA amplification occurs in the differentiated cells of the upper layers of the epithelium. Then the viral genome can be packaged and released from the surface of the epithelium. HPV triggers the DNA damage response (DDR) and employs the DDR mechanism to obtain materials necessary for viral DNA synthesis in the G2-like phase. DDR pathways, such as ataxia-telangiectasia mutated and Rad3-related pathways, are activated, and related factors are recruited to nuclear replication foci.^35,37^ These regions overlap fragile sites that are hard to replicate and susceptible to replication stress. It’s worth mentioning that HPV genome integration probably occurs in these fragile sites.^35^ Early proteins make a difference to maintaining the process of infection. The E1 and E2 proteins initiate the replication of HPV DNA and locate HPV particles to cellular chromosomes. E6 and E7 proteins maintain the life cycle of HPV replication. During the late phase of HPV replication, early proteins are increased to support the completion of this phase and facilitate the release of virions.^38^ The specific oncogenic mechanisms of early proteins of hr-HPVs are discussed in the following part.

Persistent hr-HPV infection can lead to the viral genome integrating with the host genome. Evidence has shown that the rate of genome integration is higher in HPV-16 and HPV-18 positive cancer samples than in non-cancer samples, and the integration facilitates intratumoral heterogeneity and clonal evolution.^39–41^ HPV integration mainly occurs in fragile sites that provide clonal selection advantages. HPV integration can be classified into two types. One copy is integrated in type 1 integration, while in type 2 integration, several tandem repeats are noticed. The viral genome connects to host cells mainly depend on the generation of E2 complex together with bromodomain protein 4 (Brd4), which can further activate the transcription of viral genome. In type 1 integration, despite the fact that the whole viral genome is integrated, high methylation of E2 binding sites in the p97 promoter region inhibits the expression of E2, thus reversing the inhibition of E6 and E7 and fostering the transcription of oncogenes. In type 2 integration, a combination of viral genome and Brd4 contributes to the transcription of E6 and E7. Moreover, genomic instability is caused via amplification of integration sites mediated by E1 during this process.^42^ The predominant mechanism of integration is the microhomologies-mediated DNA repair pathway. Research has demonstrated that enrichment of microhomologies is observed at or near integration breakpoints, especially when the length of microhomologies is over 4 bp. Meanwhile, the enrichment of 4-bp microhomologies remains noteworthy when the area of the flanking region extends.^43^ However, the specific progress of how microhomologies mediate the integration is still unknown. A new model has been proposed to elucidate the process of integration: synthesis‐dependent end‐joining, including junctional microhomologies, apparent blunt joining, and short insertion, which has been validated utilizing computational simulation in 4341 human-HPV junctional sequences.^39^ Notably, the hijacking of DDR to facilitate HPV DNA replication, the presence of epigenetic modification at integration sites, and the location of transcriptionally competent region within the host chromatin all contribute HPV integration, which further promotes oncogenesis.^44^

hr-HPV integration has numerous ways to alter the state of host cells, thereby promoting the initiation and development of cancers. hr-HPV integration results in the formation of fusion transcripts, which have been found in hr-HPV-related CCs. Fusion transcripts elevate the mRNA expression levels of E6 and E7 and inhibit the E2 expression, which serves as a transcriptional inhibitor of E6 and E7.^45^ The overexpression of E6 and E7 facilitates oncogenesis by disrupting the transcription of tumor suppressor genes and triggering flanking gene amplification. The subsequent overexpression of oncogenes in host cells contributes to oncogenesis.^46^ The fusion gene E1C, a representative product of HPV integration, has been confirmed to facilitate the malignant transformation of cervical cells.^47^ HPV integration contributes to the generation of extrachromosomal DNA (ecDNA), which enables cells to proliferate unlimitedly, making them promising drivers of clonal evolution.^48^ ecDNA keeps the integrity of E6 and E7 genes, which can encode oncogenic proteins and specific oncogenic signaling pathways are detailed in the following text. Moreover, together with epigenetic modification, the dysregulation of cellular genes mediated by HPV integration can cause oncogenesis as well.^49,50^ HPV integration induces chromosomal instability, rendering host cells more susceptible to cancers. Upon integration, the HPV breakpoint-induced cellular super-enhancers (BP-cSEs) are generated. BP-cSEs can interact with chromosomes and induce genomic rearrangements. Acting as ecDNA, BP-cSEs dysregulate chromosomal genes, indicating the role of HPV integration in oncogenesis.^51^ Moreover, HPV integration leads to chromosomal translocation and genomic variation, promoting clonal evolution and heterogeneity in cancers, mainly through the form of ecDNA.^40^ Research has also observed that HPV integration dysregulates local chromatin and transcriptome in HPV-positive cancers. This disruption upregulates the expression of oncogenes, thereby promoting oncogenesis by altering gene splicing, modifying the epigenome, and affecting transcriptional regulation.^52^

Moreover, dysregulated epigenetic modification, including DNA methylation, histone modification, noncoding RNA (ncRNA) regulation, and chromatin regulation, plays a key role in the occurrence and progression of cancers. Aberrant methylation of the host cell genome and HPV DNA causes the dysfunction of tumor suppressor genes, thereby accelerating the progression of cancers. WID-qCIN test, based on DNA methylation, has been developed to detect cervical intraepithelial neoplasia (CIN) and predict the development of diseases in hr-HPV-positive women.^53^ Histone modifications, like acetylation, methylation, and phosphorylation, are vital to the development of cancers. The E6 of hr-HPVs can suppress SET7-mediated methylation of p53 and downregulate histone methylation mediated by p53 coactivators, decreasing the binding of p53 to chromatin and transactivation of p53.^54^ E6 inhibits acetylation of p53 and nucleosomal core histones mediated by p300, which disrupts the activation of genes targeted by p53 and causes the development of cancers.^55,56^

HPV has various ways to evade host immune responses. Transporting viral DNA in the form of vesicles can protect HPV particles from the identification of pattern recognition receptors (PRRs), which is crucial to the persistent infection of HPVs. Early proteins of hr-HPVs can inhibit viral DNA detection by the cyclic GMP-AMP synthase -stimulator of interferon genes (cGAS-STING) pathway, thus impairing the transcription of interferons (IFNs) and IFN-stimulated genes (ISGs) by inhibiting the transcription of IFN-κ. E6 and E7 proteins of hr-HPVs also work cooperatively to suppress the generation of ISGs, thereby evading the antiviral responses of host cells. In addition, E6 and E7 proteins of hr-HPVs can dysregulate the cGAS-STING pathway and Toll-like receptor (TLR) signaling, which ultimately leads to immune evasion and persistent infection.^57,58^ Apart from innate immunity, early proteins of hr-HPVs, especially E6 and E7 proteins, affect adaptive immunity as well. hr-HPVs can impair specific human leukocyte antigen (HLA) molecules and recruit regulatory T cells to inhibit the amplification of effector T cells, therefore impeding antitumor responses.^57^ Meanwhile, the emergence of dysfunctional and exhausted T-cell subsets has been observed in HPV-positive malignancies. HPV-positive malignant cells can also generate tenascin C to interact with stroma to suppress the proliferation and activation of T cells, thus aiding hr-HPVs to evade immune responses.^59^

## Oncogenic signaling pathways of hr-HPV

**Fig. 4:**
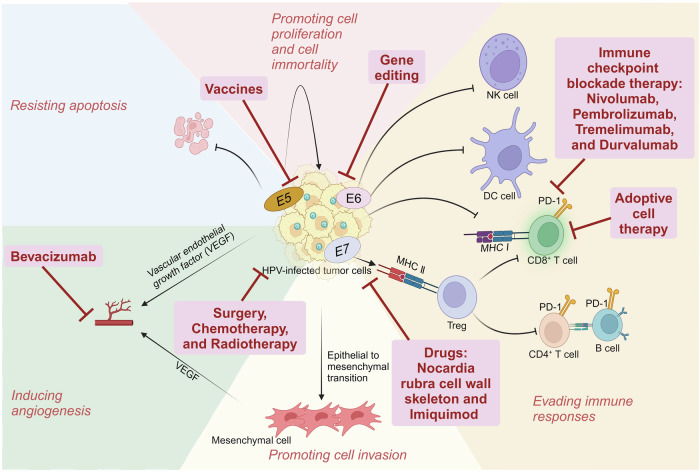
Oncogenic mechanisms of hr-HPVs and therapeutic strategies for hr-HPV-related cancers. hr-HPVs contribute to the oncogenesis through various ways, including evading immune responses, promoting cell proliferation, resisting apoptosis, inducing cell immortality, fostering angiogenesis, and promoting cell invasion. Different types of therapies treating hr-HPV-related cancers have been widely applied in clinic, aiming to gain better efficacy and prognosis. This figure was created with BioRender (www.bioender.com)

Given that persistent hr-HPV infection is a key factor in the initiation and development of cancers, oncogenic signaling pathways of hr-HPVs need to be discussed. In this review, we focus on the early proteins that hr-HPVs encode and their roles in initiating and developing cancers (Fig. 4).

### E1 and E2

The E1 protein is an adenosine triphosphate-dependent helicase with conserved structure. The E1 protein binds to the replication origin and unwinds the viral DNA to initiate DNA replication. The E1 protein consists of an N-terminal domain, a DNA binding domain, an oligomerization domain, and a C-terminal helicase domain. The N-terminal domain is responsible for transporting the E1 protein between the nucleus and the cytoplasm. The DNA binding domain is capable of identifying particular sequences and binding to the helicase domain around viral DNA. The E2 protein, existing in the form of a dimer, includes an N-terminal domain responsible for the transcriptional activation of DNA replication and a C-terminal domain that serves to bind with specific DNA. In order to initiate DNA replication, the E2 protein binds to E1 to form a complex at the replication origin. Then, the E2 protein is extracted, and the E1 protein is recruited to form double trimers. At last, the double hexamer E1 complex unwinds DNA and recruits replication factors such as topoisomerase I and DNA polymerase α-primase to initiate DNA synthesis.^60^

E1 can modulate HPV transcription epigenetically via binding to nucleosomes that are essential for regulating HPV transcription.^61^ In addition, E1 affects cell functions by dysregulating the expression of different genes. Research has verified that the overexpression of HPV-16 E1 and HPV-31 E1 suppresses the proliferation of cells by means of inducing cell cycle arrest, necrosis, or apoptosis.^62^ E1 dysregulates the expression of genes and proteins involved in transcription, apoptosis, DNA damage pathways, and immune pathways,^63^ which ultimately promotes oncogenesis. E1C, composed of an integration of the HPV-58 E1 and CMAHP gene, fosters colony formation and malignant transformation of cervical cells. Analyzed by RNA sequencing, E1C upregulates gene expression such as S1PR3, NCL, and HNRNPD, high levels of which indicate a poor prognosis of CC. Meanwhile, E1C downregulates the expression of genes, including MTRNR2L8, KRT80, and CLDN4, and the decreased expression of these genes also suggests a poor prognosis.^47^ Besides, E1 has been shown to inhibit the expression of IFNs, including IFNβ1 and IFNλ1, and activate the nuclear factor kappa-B (NF-κB) pathway, which indicates the role of E1 in immune regulation.^60,64^

Apart from being able to initiate DNA synthesis, E2 can inhibit the expression of E6 and E7 by binding to specific sites in the LCR region. Binding to Brd4, TIP60, and EP400 contributes to E2-mediated transcriptional repression of the LCR region. Demethylation of histone H3 lysine 4 is induced by the interaction between E2 and SMCX; therefore, the transcriptional activity of the LCR region is suppressed, which finally contributes to the inhibited expression of E6 and E7.^65^ E2 also blocks p97 and p105 promoters to inhibit the expression of E6 and E7.^66,67^ Disruption of E2 leads to the aberrant expression of E6 and E7, which is a precursor of oncogenesis. What’s more, E2, E6 and E7 increase the generation and secretion of interleukin 10 (IL-10) that can aid cancer cells to escape immunologic surveillance. IL-10 increases the expression of E6 and E7, which results in a vicious cycle. The overexpression of IL-10 can lead to the transformation of squamous intraepithelial lesions (SIL) to CC.^68^

### E4

The E4 open reading frame (ORF) exists in the E2 ORF. The E4 protein is generated as an E1^E4 fusion protein through mRNA splicing.^20^ The E4 protein works in the late stage of the viral life cycle. The E4 protein owns a leucine cluster motif near the N-terminus, which is necessary for its interaction with keratin. The C-terminus of the protein aids to regulate keratin, vital for viral assembly and transportation. The E4 protein can inhibit the phosphorylation of SPRK1 to modulate the distribution of E2.^69^ Research has demonstrated that the expression and accumulation of E4 can lead to G2-phase arrest, which prevents nuclear translocation and phosphorylation of proteins participating in the process of mitosis. The viral vegetative amplification is ultimately promoted, fostering persistent HPV infection.^70^

### E5

**Fig. 5:**
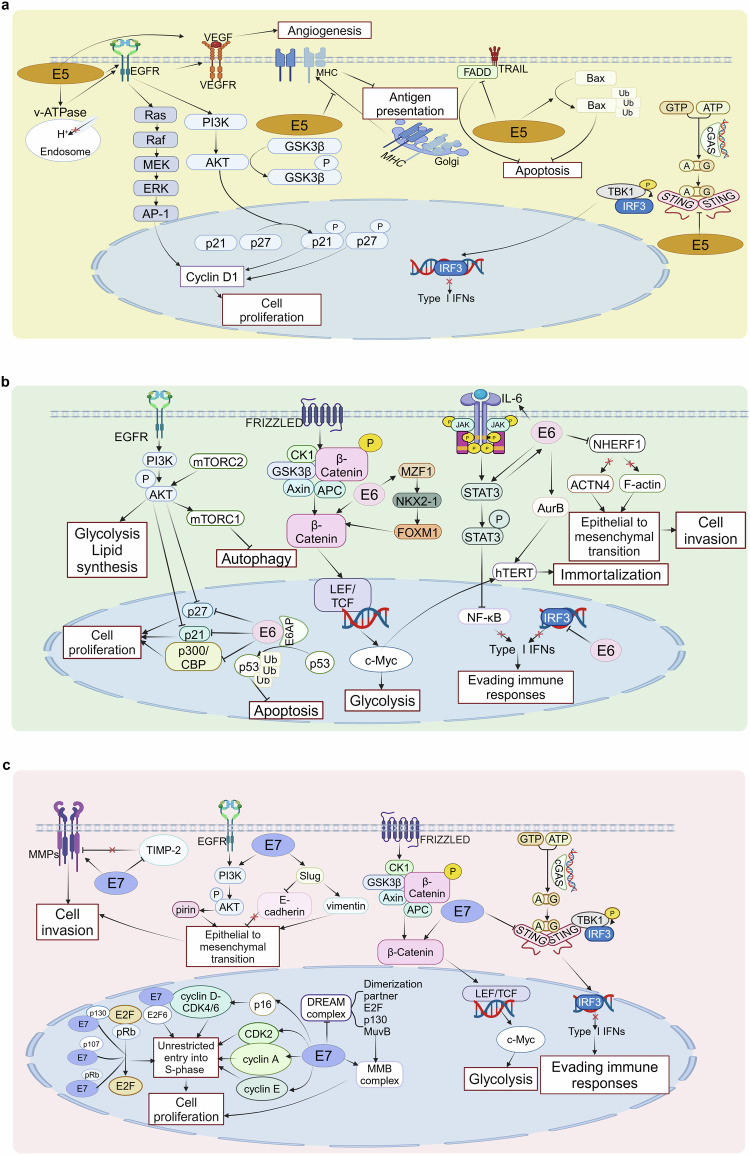
Oncogenic signaling pathways of hr-HPVs. **a** Oncogenic signaling pathways of E5. E5 can activate epidermal growth factor receptor (EGFR) signaling. Through EGFR, E5 activates the PI3K/AKT and MAPK/ERK pathways to promote uncontrolled cell proliferation. Moreover, E5 facilitates cell migration and inhibits apoptosis and immune responses, which ultimately leads to the generation of cancers. **b** Oncogenic signaling pathways of E6. E6 is well-known for binding with and degrading p53 in a ubiquitin–proteasome way. Degradation of p53 promotes cell proliferation and inhibits apoptosis. E6 is able to inhibit autophagy and immune responses and facilitate cell immortalization and invasion independent of p53 degradation as well. In addition, E6 promotes glycolysis and lipid synthesis to support cell growth. **c** Oncogenic signaling pathways of E7. E7 binds with pRb to release E2F, promoting cells entering the S-phase. E7 can increase the expression of cyclins to enable cells to alter from the G1-phase to S-phase unrestrictedly, resulting in uncontrolled cell proliferation. Moreover, E7 boosts cell migration and hinders immune responses to further stimulate the occurrence of cancers. This figure was created with BioRender (www.bioender.com)

The E5 protein, a small hydrophobic protein, comprised of three regions, an N-terminal domain, a C-terminal domain, and a central domain located between them,^20^ is characterized as sequence heterologous in different types of HPVs.^71^ Notably, only alpha-HPVs can encode the E5 protein.^72^ E5 proteins such as those derived from HPV-16 and HPV-18 exist in the endoplasmic reticulum, Golgi, endosomes, nuclear membrane, and plasma membrane.^73–75^ E5 is recognized as one of the oncoproteins and can reinforce the function of E6 and E7 in immortalizing keratinocytes.^76^ E5 is of great significance in modulating cell growth, viral replication, and various signaling pathways to initiate oncogenesis (Fig. 5a).

By means of diverse pathways, E5 is capable of modulating the proliferation, differentiation, and transformation of cells. E5 inhibits the transforming growth factor (TGF-β)/SMAD signaling, which modulates immune responses and uncontrolled cell proliferation.^77^ Moreover, E5 can interact with the epidermal growth factor receptor (EGFR) and activate pathways associated with EGFR.^78^ Through the interaction between E5 and the vacuolar ATPase (v-ATPase), the v-ATPase-mediated acidification of endosomes is disrupted, facilitating the recycling of EGFR.^79^ In addition, E5 suppresses the activity of casitas B-lineage lymphoma that can degrade EGFR via ubiquitination.^78,80^ Therefore, EGFR can persist longer. EGFR activates the phosphoinositide 3-kinase (PI3K)/protein kinase B (Akt) and the mitogen-activated protein kinase (MAPK)/extracellular regulated protein kinases (ERK) pathways. EGFR activates PI3K and mitogen-activated extracellular signal-regulated kinase (MEK) 1/2, which can phosphorate Akt and ERK1/2 respectively. Vascular endothelial growth factor (VEGF), a pivotal factor in angiogenesis, is finally upregulated.^81^ Interestingly, research has shown that the expression level of the E5 protein has no influence on the transcription of EGFR or VEGF in patients with HPV-16 or HPV-18 positive CC.^82^ Activation of MAPK sustains the expression of cyclin D1, which promotes cell proliferation. Activator protein 1 (AP-1), a dimer composed of the Fos and Jun proteins,^83^ can be activated by MAPK and possess the constitutive activity. AP-1 is highly significant for initiating and maintaining the expression of E6 and E7, and the viral expression is subsequently enhanced.^84^ Research has confirmed that the high expression of c-Jun is associated with poor prognosis in patients with HPV-16 and HPV-18 positive CC.^85^ Activated AKT phosphorylates GSK3β, while phosphorylated GSK3β contributes to the accumulation of cyclin D1. Moreover, activation of AKT leads to the phosphorylation of p21 and p27. Phosphorylated p21 and p27 accumulate in the cytoplasm and lose the ability to inhibit the activity of cyclin-dependent kinase (CDK) 4/6-cyclin D1, thus promoting cells entering the S-phase from the G-phase.^86^ In addition, the elevated expression of CDK4/6-cyclin D1 promotes E2F releasing from the RB/E2F complex, facilitating the progression of the cell cycle.^87^ Ultimately, the S-phase is prolonged, and the proliferation of basal layer cells and replication of viral genome is enhanced. Apart from regulating cell proliferation, E5 regulates the differentiation of keratinocytes located in the upper layer via keratinocyte growth factor receptor/fibroblast growth factor receptor 2b (KGFR/FGFR2b). E5 downregulates the expression of KGFR/FGFR2b and inhibits KGFR transporting to the endocytic degradative pathway, resulting in the inhibition of differentiation and regulation of suprabasal keratinocytes.^88^ Moreover, by means of downregulating the expression of KGFR/FGFR2b, E5 represses autophagy, which may cause oncogenesis.^89^

E5 can inhibit apoptosis to facilitate the survival and replication of hr-HPVs.^90^ E5 mainly induces the ubiquitination and degradation of death receptors to inhibit apoptosis.^91^ The extrinsic apoptotic pathway is initiated by the activation of cell surface death receptors such as tumor necrosis factor (TNF) receptor, TNF-related apoptosis-inducing ligand (TRAIL) receptors, and Fas. Once these receptors bind with corresponding ligands, trimerization occurs at the cell surface. Subsequently, the Fas-associated protein with death domain (FADD), pro-caspase 8, and/or pro-caspase 10 are recruited to form the death-inducing signaling complex (DISC). Pro-caspase 8 is activated and altered to caspases 3, 6, and 7.^92,93^ Apart from downregulating the expression of Fas to inhibit the recruitment of FADD, HPV-16 E5 also disrupts the formation of the TRAIL DISC and the cracking of pro-caspase 8,3, and poly ADP-ribose polymerase.^94^ HPV-16 E5 decreases the expression of Bax and Bak through increasing the proteasome-ubiquitin degradation of Bax. Meanwhile, E5 increases the expression of Bcl-2.^95^ As a result, apoptosis is suppressed.

E5 plays an essential role in evading immune responses. E5 is able to downregulate the expression of major histocompatibility complex (MHC) I, which is important for antigen presentation and subsequent induction of immune responses. Via inhibiting compartment acidification, E5 mediates the retention of MHC I molecules in the Golgi and decreases the expression of MHC I on the membrane surface. Moreover, by inhibiting MHC II transporting to the cell surface, E5 disturbs the expression and stability of MHC II. Through binding with MHC molecules and further suppressing their transportation to the plasma membrane, E5 hinders antigen processing and promotes immune evasion.^96,97^ CD1d, which presents antigens to natural killer T cells, is downregulated by E5 and is incapable of being transported to the surface of cells, thus successfully evading immunologic surveillance.^98,99^ E5 directly binds with mitochondrial antiviral-signaling protein and STING to inhibit the cGAS-STING and retinoic acid-inducible gene-1 (RIG-1)/melanoma differentiation-associated gene 5 pathways, ultimately eliminating immunologic surveillance.^100^

E5 upregulates the expression of paxillin and downregulates the expression of A/C protein; thereby altering cell migration.^101,102^ What’s more, E5 may modulate cell adhesion and cell migration via matrix-metalloproteinases (MMPs). MMPs pave the way for cell migration due to the ability to degrade the basement and extracellular matrix and separate cells from the extracellular matrix and adjacent cells.^103^ HPV-16 E5 promotes cell migration, and accelerates the transition to a malignant phenotype by downregulating the expression of E-cadherin.^104^ Nevertheless, the detailed underlying mechanisms need to be further studied.

E5 is involved in metabolic reprogramming and aims to provide energy and environment conducive to the proliferation of cancer cells. Elevated expression of EGFR activates the MAPK/ERK, Janus kinase (JAK)/signal transducer and activator of transcription (STAT), PI3K/Akt, and protein kinase C pathways, contributing to increased glycolysis, lipid synthesis, and inhibited glycogen synthesis.^105^

### E6

E6, composed of around 150 amino acids, includes two zinc finger-binding domains flanked by four Cys–X–X–Cys (CXXC) motifs. Possessing a C-terminal PDZ (PSD-95/DLG/ZO-1) binding motif (PBM) is a unique characteristic of hr-HPVs. PBM is essential for the oncogenesis of the E6 protein of hr-HPVs.^106^ This motif facilitates the binding of E6 to proteins containing PDZ domains, thus influencing the generation of cancers. E6-associated protein (E6AP), which owns a PDZ structure, is well-known for interacting with E6 and forming a heterodimer. The interaction between E6 and the LXXLL motif of E6AP is a prerequisite for the fabrication of E6/E6AP/P53 complex.^107^ Degradation of p53 in a ubiquitin–proteasome way, a marker that indicates the activity of E6, causes genomic instability,^108,109^ which is closely linked with oncogenesis. Here, we summarize mechanisms through which E6 induces oncogenesis (Fig. 5b).

E6 is capable of triggering uncontrolled cell proliferation. Research has verified that the proliferation of HPV-positive cells can be inhibited via downregulating the expression of E6.^110,111^ p53 suppresses cyclin B, leading to G2/M-phase arrest and cell cycle regulation.^112^ By degrading p53, the mentioned functions can be reversed. HPV-16 E6 inhibits the coactivation of p300/CBP, leading to uncontrolled cell proliferation.^113^ E6 impairs cell cycle checkpoints by downregulating the expression of CDKs and cyclin inhibitor kinases such as p21 and p27, thus causing uncontrolled proliferation.^114^ E6 inhibits p21 and p27 probably by degrading p53 and upregulating c-Myc, which needs to be further studied.^115^ E6 activates the PI3K/Akt/mammalian target of rapamycin (mTOR) pathway. The mTOR complex (mTORC) 2 phosphorylates Akt, which induces the activation of mTORC1. mTORC1 promotes viral replication and cell proliferation, and inhibits autophagy.^116,117^ Research has verified that inhibiting the PI3K/Akt/mTOR pathway can further inhibit cell proliferation.^118,119^ E6 inhibits the expression and nuclear import of STAT1 to promote the amplification of the viral genome, thereby maintaining the low-level replication of the viral genome.^120–123^ E6 further enhances STAT3 signaling by inducing IL-6 expression through the Ras-related C3 botulinum toxin substrate 1/NF-κB pathway. STAT3 can in turn promote the expression of E6.^124,125^ Activated STAT3 is able to promote the transcription of genes involved in cell proliferation.^126^ STAT3 colocalizes with AP-1 components and interacts with AP-1, which further influences the cell cycle and proliferation of cells.^85^ Nevertheless, recent research has found that there’s no obvious difference in the proliferation rates of cells between STAT3 KO CC cells and their parental counterparts. Although ruxolitinib effectively inhibits the phosphorylation of STAT3, it shows no effect in the proliferation or growth of cells,^127^ which needs further research. Mastermind like transcriptional coactivator 1 (MAML1), a coactivator of NOTCH, interacts with E6 and stabilizes E6. A synergistic effect between E6AP and MAML1 fosters cell proliferation.^128^ Research has confirmed that E6 increases the expression of NOTCH1 and elevates NOTCH signaling to promote cell proliferation.^129^ However, the distinct mechanism still needs to be studied. Na^+^ /H^+^ exchanger regulatory factor-1 (NHERF1) can be degraded by E6 in an E6AP-dependent proteasome manner. The degradation of NHERF1 activates the canonical Wnt/β-catenin pathway.^130^ E6 induces the expression of MZF1 and the subsequent transcription of NKX2-1 and FOXM1. FOXM1 enhances the transcription and translocation of β-catenin.^131^ Besides, HPV-18 E6 increases the transcription of β-catenin and T-cell factor 4, which further activates the Wnt/β-catenin pathway and promotes the expression of targeted genes such as c-Myc and cyclin D1.^132^ The activated Wnt/β-catenin pathway cooperatively works with E6 to enhance cell proliferation.^133^ Notably, extracellular vesicular Wnt7b, upregulated by E6 in HPV-16 and HPV-18 positive cells, can activate β-catenin signaling to promote angiogenesis and cancer invasion.^134^ Via increasing SNAT1, a glutamine transporter, E6 further elevates intracellular levels of glutamine and glutamate, which provides abundant energy to support uncontrolled cell proliferation.^135^ RBM15, which determines N6 methyladenine (m6A) modification, is upregulated and accumulated in CC cells through inhibition of E6-mediated degradation. RBM15 promotes the proliferation and growth of CC cells through m6A modification. Furthermore, RBM15 increases the expression of c-Myc, which promotes cell proliferation and oncogenesis.^136^ Upregulated c-Myc suppresses miR-146a-5p promoter to inhibit the expression of miR-146a-5p. Meanwhile, KDM2B is upregulated by E6, which in turn elevates the expression of E6. Decreased miR-146a-5p and increased KDM2B work synergistically to promote cell proliferation and the progression of cell cycle.^137^ Moreover, p53 degradation contributes to the downregulation of miR-22 and miR-34a, which subsequently promotes cell proliferation and migration.^138^

The presence of PBM region is a unique feature of E6-induced oncogenesis. E6 regulates tumor invasion via binding to proteins containing the PDZ motif.^106^ Research has confirmed that PBM of HPV-16 contributes to the migration and invasion of cancer cells.^139^ The Scribble complex, the Crumbs complex, and the Par complex, all of which possess a PDZ motif, play pivotal roles in maintaining cellular polarity.^140^ Membrane-associated guanylate kinase 1 (MAGI1) can serve as a tumor suppressor in the progression of cancer. E6 targets and degrades MAGI1, disrupting cell adhesion and thereby promoting the invasion and metastasis of cancer cells.^141,142^ Once binding with E6, the mentioned complexes fail to maintain contact between cells and cellular polarity. Loss of polarity may lead to abnormalities in signal transportation, cell transformation, the occurrence of epithelial to mesenchymal transition (EMT), and ultimately the progression and invasion of cancer.^106^ NHERF1 is downregulated by HPV-16 E6, leading to the upregulation of ACTN4 and F-actin. Actin cytoskeleton assembly and cancer cell migration are thus promoted.^139^ EMT is well recognized to be of vital importance in cancer metastasis. E6 has been found to upregulate EMT levels and stemness of HPV-positive CC cells. As prominent markers of EMT, the mRNA expressions of N-cadherin and vimentin are significantly elevated in C33A cells. The overexpression of E6 can increase the sphere-forming activity of C33A cells and mRNA expression of OCT4, Nanog, SOX4, and other stemness-related transcription factors.^143^ Consequently, E6 promotes the invasion and migration of cancer cells, resulting in malignant phenotypes.

E6 is able to inhibit apoptosis in various ways. p53 is well-known to trigger apoptosis in cells with DNA damage and interacts with pro-apoptotic genes such as Puma, Bax, and Noxa. Moreover, p53 interacts with and suppresses anti-apoptotic genes such as BCL-2 and BCL-xL, inducing mitochondrial outer membrane permeabilization, thereby causing mitochondrial apoptosis in a transcription-independent manner.^144,145^ E6 reverses the function of p53 by binding to and degrading p53. E6 degrades FADD and pro-caspase 8 to inhibit the formation of DISC and the subsequent activation of caspases 8, 3, and 7. Therefore, TRAIL-mediated apoptosis is inhibited independently of p53.^146^

Through interacting with TYK2, E6 inhibits the phosphorylation of STAT1 and STAT2 and the subsequent transcription of ISGs. The impaired ISGs signaling disrupt the production of chemokines and antigen-presenting molecules such as MHC, leading to inadequate antiviral responses and the initiation of cancers.^147^ E6-mediated STAT3 upregulation promotes tumor growth by suppressing immune responses that are partially dependent on the NF-κB pathway. Studies have shown that inhibition of STAT3 leads to increased phosphorylation of p65 and NF-κB, as well as enhanced infiltration of T cells, resulting in impaired tumor growth and robust immune responses.^148^ Owing to the phosphorylation, transcriptional activity, and translocation ability of interferon regulatory factor 3 being inhibited by its binding with E6, the downstream expression of IFNβ is suppressed, which facilitates HPV to evade immune responses.^149^

Immortalization of cells is a hallmark of HPV-induced cancer progression. E6 activates human telomerase reverse transcriptase (hTERT) via several mechanisms: promoting the expression of c-Myc, recruiting the transcriptional factor Sp1, downregulating NFX1-91, and increasing telomerase activity, which collectively promote the proliferation and immortalization of cells.^150–152^ NFX1-123, highly expressed in CC, interacts with HPV-16 E6 and synergistically increases the activity of hTERT and telomerase.^153^ E6 increases the expression of AurB, and the interaction between AurB and E6 further enhances the level of hTERT, which results in cell immortalization.^154^

Degradation of p53 decreases the expression of TP53-induced glycolysis and apoptosis regulators, which leads to increased glucose transporters-1 (GLUT-1), GLUT-2, and glycolysis. Degraded p53 increases the level of hypoxia-inducible factor-1α (HIF-1α), promoting glycolysis, lipid uptake, and synthesis.^155^ Besides, elevated expression of c-Myc upregulates enzymes involved in glycolysis and fosters glycolysis.^156^ Meanwhile, E6 activates the PI3K/Akt/mTOR pathway to facilitate glycolysis and lipid synthesis.^105,157,158^

### E7

E7, a small protein of around 100 amino acids, is composed of conserved regions 1/2/3 (CR1/2/3). The sequences of a tiny part of CR1 and almost total CR2 are similar to the adenovirus E1A protein and a large T antigen of SV40.^159^ Positioned at the C-terminal end, CR3 consists of conserved sequences and encodes a zinc finger domain, which includes two CXXC motifs separated by 29 amino acid residues.^160^ E7 disturbs DNA damage repair, resulting in the accumulation of DNA lesions and the occurrence of homologous recombination deficiency.^161^ E7 directly interacts with and regulates RING finger protein 168, which is essential for viral replication. Through the interaction, E7 suppresses DNA double-strand break signaling, promoting the occurrence of genomic instability and the development of cancers.^162^ Due to its ability to trigger DNA damage and genomic instability, E7 is considered as an oncoprotein that promotes the generation and progression of cancers (Fig. 5c).

E7 is widely known for binding with pRb to disturb the cell cycle and promote uncontrolled cell proliferation. Serving as a cell cycle regulator, pRb can interact with E2F to strictly modulate the transition of cells into the S-phase. In late G1-phase, cyclin E-CDK2 phosphorylates pRb to dissociate from E2F, thereby facilitating the transition of the cell cycle into the S-phase.^163,164^ Cyclin Y-CDK4 and cyclin D-CDK4/6 phosphorylate pRb to release E2F, enhance the transcriptional activity of E2F, and promote the G1/S-phase alternation.^165,166^ E7 competitively binds with and degrades pRb, as well as related proteins p107 and p130, resulting in the release and increased activity of E2F. Interestingly, a study has revealed that inhibition of HPV-18 E7 mainly leads to the elevated expression of pRb in HeLa cells, while the expression of pRb differs insignificantly in EC109, EC9706, and Tca83 cell lines, which indicates that E7 tends to interact with different targets in diverse cancer cells.^167^ The expression of cyclin A and cyclin E is subsequently increased and promotes the transition into the S-phase.^168^ E7 interacts with p21 and p27 to reverse the inhibition of CDK2, thus facilitating the transition into the S-phase.^169,170^ Moreover, E7 targets p21 and p27 to inhibit apoptosis.^171^ E7 can directly bind with E2F6, a member of the E2F family that can repress transcription, causing the extension of the S-phase.^172^ The DREAM complex, composed of dimerization partner, Rb-like, E2F, and MuvB, functions as a transcriptional repressor. By dissociating MuvB from p130, E7 disrupts the assembly of the DREAM complex and promotes the formation of the Myb-MuvB (MMB) complex, which is necessary for G2/M-phase gene expression. Genes targeted by the DREAM and MMB complex are upregulated, and cell proliferation is thus boosted.^173^ E7 induces the expression of H3K27-specific demethylases KDM6A and KDM6B and promotes cell growth in HPV-16 positive cells. Moreover, p16, a marker of hr-HPVs, is induced by KDM6A and KDM6B.^174^ E7 upregulates and activates miR-106a in HPV-16-positive CC cells. miR-106a further regulates the AMPK/mTOR pathway and increases the expression of phosphorylated mTOR via targeting liver kinase B1, which ultimately leads to uncontrolled cell proliferation and inhibited autophagy.^175^ E7 cooperates with E6 to upregulate miR-18a, which inhibits the STK4-Hippo pathway and facilitates cell proliferation in HPV-positive cells.^176^ HPV-16 E7 decreases the phosphorylation level of AKT, p70 S6K, and 4E-BP1, accompanied by increased internal ribosomal entry site-dependent translation, which increases the expression of c-Myc and BAX, leading to cell proliferation and cancer progression.^177^

Cell invasion and metastasis play pivotal roles in the progression of cancer. MMPs can degrade the basal membrane and extracellular matrix to promote cell invasion. Research has confirmed that high expression of MMPs, especially MMP-2, and tissue inhibitors of metalloproteinase-2 (TIMP-2) is closely associated with high aggressiveness and poor prognosis.^178^ HPV-16 E7 upregulates the activity of MMP-9 and downregulates proteins, including cysteine-rich proteins with kazal motif gene and TIMP-2, which inhibits the activity of MMP-9, thus contributing to cell invasion and tumor metastasis.^179^ HPV-18 E7 synergistically enhances the expression of MMPs in conjunction with H-rasV12, mediated by the MEK/ERK pathway. Therefore, the upregulated MMPs enable cells to migrate and invade in a manner unrelated to the degradation of pRb.^180^ HPV-16 E7 and E6 synergistically induce the accumulation of β-catenin in the nucleus, increase the expression of c-Myc and transcription factors, and promote the occurrence of EMT.^181^ In addition, E7 also induces the activity of fibroblast growth factors, which enhances the invasive ability of cancer cells.^182^ By interacting with gelsolin, HPV-16 E7 promotes EMT via cytoskeletal actin remodeling. Moreover, the interaction between E7 and gelsolin leads to actin polymerization and accumulation of phosphorylated YAP in the cytoplasm, which may contribute to EMT.^183^ By increasing c-Myc, HPV-16 E7 and E6 upregulate SNHG12, which can further upregulate the expression of Slug, a transcriptional repressor of E-cadherin, and ultimately contribute to the occurrence of EMT.^184^ E7 can directly upregulate the expression of Slug, which decreases the expression of E-cadherin and increases vimentin expression simultaneously, contributing to EMT and migration.^185^ Through activation of the PI3K/AKT1 pathway, HPV-16 E7 overexpresses pirin, which promotes cell migration and EMT in CC and oral cancer cells.^186,187^ Moreover, research has confirmed that HPV-16 E7 and E6 work cooperatively to decrease the expression of miR-23b-3p, thereby enhancing the occurrence of EMT and promoting the invasion and migration of CC cells.^188^

E7 upregulates H3K9-specific methyltransferase and SUV39H1, which leads to the formation of heterochromatin at the promoter regions of RIG-1, cGAS, and STING. The activation of the RIG-1 and cGAS-STING pathways is thus suppressed, and the synthesis and release of IFNs are inhibited as well, causing immune evasion from the host immune system.^189^ HPV-16 E6 and E7 downregulate the expression of TLR9 at mRNA and protein levels,^190^ inhibiting the generation of IFNs mediated by NF-κB and IRF7.^191^ HPV-16 and HPV-18 E7 downregulate the expression of miR-142-5p that directly targets PD-L1. PD-L1 is thus enhanced, and immune responses are suppressed.^192^ HPV-16 E7 negatively regulates the expression of MHC I, inhibits antigen processing, and simultaneously hinders T-cell-mediated immune responses.^193,194^ HPV-16 E7 impedes the formation and translocation of interferon-stimulated gene factor 3, which induces the transcription of ISGs. Therefore, further synthesis of IFNs is suppressed.^184^

Telomerase activity, together with the inactivation of pRb and p16, results in the immortalization of cells.^195^ hr-HPV E7 is capable of degrading PTPN14, contributing to the limited differentiation and increased immortalization of keratinocytes.^196^ HPV-16 E7 is capable of inhibiting the activity of TNFα-induced NF-κB due to its integrity of pRb binding sites. The inhibition of NF-κB promotes the immortalization of cells, especially in the cervical transformation zone.^177,197^

E7 is able to alter the metabolism of cells to facilitate the development of cancers as well. HPV-16 and HPV-18 E7 and E6 upregulate IGF2BP2 to increase the expression of c-Myc by identifying m6A-modified sites, which can increase aerobic glycolysis in CC cells.^156^ Besides, E7 activates the PI3K/Akt/mTOR pathway and upregulates the expression of HIF-1α, causing enhanced glycolysis and lipid synthesis.^105^

## hr-HPV as a target used in the clinic

Since hr-HPV plays an irreplaceable role in the generation and progression of cancers, the detailed applications of hr-HPV in screening, diagnosis, and treatment need to be discussed. Currently, the application of hr-HPV, especially in CC, is widely recognized and has benefited a large number of individuals suffering from persistent hr-HPV infection.

### Value of hr-HPV in prevention and screening

Unless detected and treated as early as possible, cells that experience persistent hr-HPV infection can progress from low-grade SIL (LSIL) into high-grade SIL (HSIL), which are prone to oncogenesis. Hence, prevention and timely screening are of vital significance.

The cervical HPV test has 90% sensitivity for detecting precancer. HPV screening combined with cytology test is recommended once every 5 years for individuals aged 30–65 years.^198^ The possibility of evolving into CC will be less than 0.15% within 5 years if the HPV test result is negative.^199^ If the HPV test result is negative, re-examination is recommended once every 3 or 5 years. Considering the positive HPV test result together with LSIL for cytology test, annual re-examination is recommended, along with a colposcopy. If the HPV test result is positive and the cytology result is HSIL, colposcopy is required and subsequent treatment, such as ablative treatment, should be considered. Additionally, expedited treatment is highly recommended if the risk of CIN3 is greater than 60% (e.g., HPV-16-positive accompanied by HSIL).^199,200^ Apart from testing genotypes of HPV, the p16/Ki67 dual-stain test is also applied to further predict the risk of precancer and confirm the necessity of colposcopy.^201^ HPV DNA combined with DNA methylation detection, which can indicate CIN2/3 and CC, shows promise for the future of CC screening. Research has shown that the result of HPV DNA test together with DNA methylation test from urine is highly consistent with the result from cervical scrapes.^202^ Due to the easy access to urine samples, this approach may be more beneficial for the promotion of CC screening.

HNSCC tends to be diagnosed late, so new methods to timely detect HNSCC need to be developed. hr-HPV serological tests have been widely studied. Predictive models incorporating hr-HPV serological tests are of great convenience when tumor tissue is unavailable and of predominant sensitivity, specificity, and accuracy. A new predictive model for HNSCC using hr-HPV serostatus, genetic factors, and risk factors has been developed in Europe. The accuracy of prediction for OPSCC is over 90% when hr-HPV serostatus is included.^203^ A newly clinico-serological predictive model has been developed and has shown its superiority in predicting hr-HPV status and prognosis in OPSCC compared with p16 tissue standard. Moreover, researchers have found that HPV-16-related OPSCC tends to match the model best.^204^ However, hr-HPV serological tests have not been widely applied clinically. In terms of feasibility, specificity, sensitivity, and accuracy, models incorporating hr-HPV serological tests are promising in the screening of HNSCC.

Despite the fact that HPV DNA test has been widely applied for cervical cancer screening in clinic, limitations still exist. Although the HPV DNA test is considered to be a sensitive screening tool, it also produces substantial false-negative results. There are several possible explanations for these false-negative results: on one hand, it may result from the small lesion size with a few abnormal cells or the presence of genotypes not covered by HPV test; on the other hand, the integration of the virus inside the host cell could significantly hinder HPV detection by reducing the viral load at the surface of lesions.^205–207^ Besides, in LMICs with limited number of well-trained health workers and financial resources, people may have no access to HPV screening, which greatly limits its generalizability. Moreover, variations in populations and testing methods may lead to bias in the sensitivity and specificity of HPV DNA test results.^208^ Therefore, making these techniques more convenient, cheaper and stable to benefit people around the world may be the future focus.

Prophylactic vaccines have been developed and can prevent the occurrence of CC, HNSCC, anal cancer, penile cancer, vulvar cancer, and vaginal cancer to a certain extent. Data has shown that the effectiveness of vaccination decreases with increasing age,^209^ and the incidence of cancers remarkedly decreases with the widespread access to HPV vaccines.^210^ Prophylactic vaccines are based on virus-like particles derived from the L1 protein and can prevent infection through the generation of L1-specific neutralizing antibodies.^211^ Nowadays, different types of prophylactic vaccines have been applied since 2006 (Table 2). Gardasil, approved by the Food and Drug Administration (FDA) for use in women aged 9–26 in 2006, is the first generation of vaccines targeting HPV-16/18/6/11. Cervarix is a bivalent vaccine targeting HPV-16/18, approved by the FDA for use in women aged 9–25 in 2009. Gardasil-9 is a nonavalent vaccine targeting HPV-16/18/31/33/45/52/58/6/11, approved in 2014.^212^ Xiamen Innovax Biotech has developed a bivalent vaccine targeting HPV-16/18, Cecolin, which has been approved in China in 2019. It can be applied to women aged 9–45. Based on Cecolin, a new nonavalent vaccine named Cecolin-9 has been developed, and a phase II clinical trial has been conducted to validate its effectiveness (NCT03935204).^213^ The Serum Institute of India has developed a quadrivalent HPV vaccine called Cervavac targeting HPV-16/18/6/11. The vaccine is suitable for people aged 9–26 regardless of gender. The vaccine has completed its phase III clinical trial and has been approved by India in 2022.^214^ A bivalent vaccine targeting HPV-16/18 developed by Shanghai Zerun, which has completed a phase III clinical trial (NCT01548118), has been approved by China in 2022. Despite the fact that vaccines based on L2 show lower immunogenicity and lower levels of neutralizing antibodies than L1-based vaccines, L2-based prophylactic vaccines are still promising. Chimeric L1-L2 virus-like particles and vaccines combining L2 with E6 and/or E7 proteins may be wonderful strategies to enhance the prophylactic efficacy of L2-based vaccines.^215,216^ Prophylactic vaccines have been validated to be effective in clinical trials and nearly have no serious adverse events (SAEs).^217,218^ Cervarix has been confirmed to be effective and titers of antibodies remain high 9.4 years after the first vaccination. In various studies, Gardasil has been verified to be effective against HPV-16/18-related CIN2/3 or worse lesions and titers of antibodies remain high especially against HPV-16 5 years postvaccination.^219^ Notably, 5 years after vaccination, compared with Cervarix, Gardasil has lower levels of geometric mean titers (GMTs) against HPV-16 and HPV-18. Meanwhile, in terms of geometric mean number of CD4^+^ T cells against HPV-16 and HPV-18 induced by vaccination, Cervarix is better than Gardasil.^220^ Gardasil-9 has been confirmed to be effective against CIN2/3 8 years after vaccination.^221^ However, almost 20% of recipients have a loss of anti-HPV-18 titers and 15% of recipients have a loss of anti-HPV-45 titers 2 years after vaccination of Gardasil-9. Besides, anti-HPV-31, 33, 52, and 58 titers can decline 2 years after vaccination.^212^ Since there are difficulties in the completion of three doses of vaccines, the WHO has recommended a single dose to replace multiple doses in order to better promote the spread of vaccines. A meta-analysis has observed that compared with 3 doses of Gardasil, 2 doses of Gardasil have inferior antibody responses against HPV-18 in 18 months. While two doses of Gardasil-9 have higher peak GMTs at 12-month interval and final protection is irrelevant to the time interval of the second Gardasil-9 dose, which makes vaccination more practical in clinic.^212^ Moreover, research has confirmed that a single dose of quadrivalent vaccine still has high titers of antibodies ten years after vaccination.^222^ Given the convenience and lower cost, a single dose of vaccine may be a trend. Due to the high price of vaccines, a large number of people from LMICs have no access to vaccines. Therefore, the incidence of hr-HPV-related cancers remains high in LMICs. More investment and lower costs of vaccines are urgently needed to be addressed in order to facilitate the spread of vaccines worldwide.^25,223^

**Table 2:** Prophylactic vaccines targeting hr-HPV

Name	Valents	Targets	Adjuvants	Expression system	Current progress	Eligible age and sex	References
Gardasil	4	HPV-16/18/6/11	Amorphous aluminum hydroxy phosphate sulfate	Recombinant Saccharomyces cerevisiae, Yeast	Approved in 2006	Females aged 9–26	^211^
Cervarix	2	HPV-16/18	3-0-Desacyl-4′-monophosphoryl lipid A, Aluminum hydroxide salt	Trichoplusia ni insect cells, Baculovirus	Approved in 2009	Females aged 9–25	^216^
Gardasil-9	9	HPV-16/18/31/33/45/52/58/6/11	Amorphous aluminum hydroxy phosphate sulfate	Recombinant Saccharomyces cerevisiae, Yeast	Approved in 2014	Females aged 9–45	^212^
Cecolin	2	HPV-16/18	Aluminum hydroxide	Escherichia coli	Approved in 2019	Females aged 9–45	^277^
Cecolin-9	9	HPV-16/18/31/33/45/52/58/6/11	Aluminum hydroxide	Escherichia coli	phase II clinical trial completed	Females aged 9–45	^213^
Cervavac	4	HPV-16/18/6/11	Aluminum based	Hansenula polymorpha	Approved in 2022	Females and males aged 9–26	^214^
Walvax recombinant HPV vaccines	2	HPV-16/18	Aluminum phosphate	Pichia pastoris, Yeast	Approved in 2022	Females aged 9–30	^278^

### Therapeutic advances in vaccines

Conventional therapy, such as surgery, radiotherapy, and chemotherapy, has been widely utilized in clinic. Given the essential role of hr-HPV in the initiation and progression of cancers, various vaccines targeting hr-HPV, expected to induce intense immune responses, have been developed to treat cancers. To date, four types of vaccines, including vector-based vaccines, peptide- or protein-based vaccines, whole-cell vaccines, and nucleic acid vaccines, have been developed and tested in clinic (Table 3).

**Table 3:** Therapeutic vaccines targeting hr-HPV

Types	Name	Targets	Highest status	Eligible population	Trial number	Efficacy	Safety	References
Vector-based	ADXS11-001	HPV-16 E7	Phase III clinical trial	Patients with high-risk locally advanced CC	NCT02853604	12-month OR rate: 34.9%, 18-month OR rate: 24.8%	3 vaccine-related serious adverse events (SAEs)	^224^
	GLBL101c	HPV-16 E7	Phase IIB clinical trial	Patients with CIN2 and HPV-16 infection	UMIN000001686	No significant difference between placebo and GLBL101c group	No vaccine-related SAEs	^225^
	IGMKK16E7	HPV-16 E7	Phase I/II clinical trial	Patients with CIN2-3	jRCT2031190034	complete response rate: 31.7% in high-dose group	No vaccine-related SAEs	^227^
	MVA-E2	HPV-16 E2	Phase III clinical trial	Patients with HPV intraepithelial lesions	–	90% overall efficacy	No vaccine-related SAEs	^279^
	TA-HPV	HPV-16/18 E6/E7	Phase II clinical trial	Patients with early CC	NCT00002916	83% overall efficacy	No vaccine-related SAEs	^280^
	TG4001	HPV-16 E6/E7	Phase II clinical trial	Patients with HPV-16-positive recurrent or metastatic malignancies	NCT03260023	48% overall efficacy	No vaccine-related SAEs	^281,282^
	Vvax001	HPV-16 E6/E7	Phase II clinical trial	Patients with HPV-16 induced CIN3	NCT06015854	HPV-16 specific T-cell response rate: 83%	No vaccine-related SAEs	^283^
	PRGN-2009	HPV-16/18 E6/E7	Phase II clinical trial	Patients with recurrent or metastatic CC	NCT06157151	Not reported	Not reported	^284^
			Phase II clinical trial	Patients with HPV-associated oropharyngeal cancer	NCT05996523			
	HB-201	HPV-16 E6/E7	Phase I/II clinical trial	Patients with HPV-associated squamous carcinoma	NCT04180215	ORR: 18%, disease control rate: 73%	No vaccine-related SAEs	^285^
	HB-202	HPV-16 E6/E7	Phase I/II clinical trial	Patients with HPV-16-positive OPSCC	NCT05108870	Not reported	Not reported	^285^
Peptide- or protein-based	ISA101	HPV-16 E6/E7	Phase II clinical trial	Patients with HPV-16-positive solid tumors	NCT02426892	ORR: 33%	2 vaccine-related SAEs	^231^
	PepCan	HPV-16 E6	Phase II clinical trial	Patients with cervical HSIL	NCT02481414	Overall histological regression rate: 45%	No vaccine-related SAEs	^232^
			Phase I/II clinical trial	Patients with HNSCC	NCT03821272	Not reported	Not reported	
	TVGV-1	HPV-16 E7	phase IIA trial	Patients with HPV-induced cervical HSIL	NCT02576561	Not reported	Not reported	^233^
	TA-CIN	HPV-16 E6/E7/L2	Phase I clinical trial	Patients with HPV-16-associated CC	NCT02405221	HPV-16 specific T-cell response rate: 78%	No vaccine-related SAEs	^286^
	PDS0101	HPV-16 E6/E7	Phase II clinical trial	Patients with locally advanced CC	NCT04580771	Not reported	Not reported	^287^
			Phase II clinical trial	Patients with recurrent and/or metastatic HNSCC and HPV-16 infection	NCT04260126			
	DPX-E7	HPV-16 E7	Phase I/II clinical trial	Patients with HPV-16-associated cancers and HLA-A*02 positive	NCT02865135	Not reported	Not reported	^288^
Whole-cell-based	DC pulsed vaccines	HPV-16 E7	Phase I clinical trial	Patients with recurrent CC	NCT00155766	Not reported	Not reported	
	DC pulsed vaccines	HPV-16/18 E7	Phase I clinical trial	Patients with HPV-16/18-infected stage IB or IIA CC	_	Intense cellular immune responses are induced.	Not reported	^243^
	TCR-T-cell vaccines	HPV-16 E7	Phase II clinical trial	Patients with HPV-associated cancers	NCT05686226	Not reported	Not reported	^235^
Nucleic acid-based (DNA-based)	VGX-3100	HPV-16/18 E6/E7	Phase IIb clinical trial	Patients with cervical HSIL	NCT01304524	Histopathological regression rate: 80%	No vaccine-related SAEs	^247,248^
			Phase I/II clinical trial	Patients with HPV-associated HNSCC	NCT02163057	Not reported	Not reported	
	pNGVL4a-Sig/E7(detox)/HSP70	HPV-16 E7	Phase I clinical trial	Patients with HPV-16-positive CIN2/3	NCT00788164	Histopathological regression rate: 33%	No vaccine-related SAEs	^249^
	pNGVL4aCRTE6E7L2	HPV-16 E6/E7/L2	Phase I clinical trial	Patients with HPV-16-positive cervical HSIL	NCT03913117	Not reported	Not reported	
	pNGVL4a-CRT/E7	HPV-16 E7	Phase I clinical trial	Patients with HPV-16-positive CIN2/3	NCT00988559	Histopathological regression rate: 30%	No vaccine-related SAEs	^289^
	INO-3112	HPV-16/18 E6/E7	Phase I/II clinical trial	Patients with CC after chemoradiation for newly diagnosed disease or therapy for recurrent and/or persistent disease	NCT02172911	Progression-free survival at 12 months rate: 88.9%	No vaccine-related SAEs	^251,290^
			Phase I/II clinical trial	Patients with HPV-associated HNSCC	NCT02163057	ORR: 27.6%	1 vaccine-related SAEs	
	GX188E	HPV-16/18 E6/E7	Phase I clinical trial	Patients with HPV-16 and/or 18- positive CIN3	NCT01634503	Complete response rate: 78%	No vaccine-related SAEs	^253,254,291^
			Phase II clinical trial	Patients with advanced, resectable HPV-16 or 18-positive HNSCC	NCT05286060	Not reported	Not reported	
	AMV002	HPV-16 E6/E7	Phase I clinical trial	Patients with HPV-associated OPSCC	ACTRN12618000140257	E6 or E7 specific T-cell immune responses rate: 83%	No vaccine-related SAEs	^292^

#### Vector-based vaccines

Usually, bacterial and viral vectors are employed as vector platforms to deliver hr-HPV-specific antigens. Vector-based vaccines possess intense immunogenicity and are capable of inducing innate and adaptive immune responses. However, high immunogenicity may be harmful for patients with low immunity. Furthermore, immune responses targeting vectors may exceed responses targeting hr-HPV-specific antigens, which is an obstacle to the use of vector-based vaccines.

Among bacterial vectors, live-attenuated listeria monocytogenes (Lm) is considered as an effective vector due to its ability to present antigens, induce antigen-presenting cell maturation, and trigger immune responses. ADXS11-001, a vaccine composed of HPV-16 E7 fused with non-hemolytic listeriolysin O, utilizing Lm as a carrier, is a therapeutic vaccine that is expected to induce both CD4^+^ and CD8^+^ T-cell immune responses. A phase I clinical trial was conducted in 2009. In 2014, 110 patients with recurrent or refractory CC following chemotherapy and/or radiotherapy were recruited, and a phase II clinical trial was conducted to study the effectiveness of ADXS11-001 with or without cisplatin. The 12-month overall survival (OR) rate was 34.9%, and the 18-month OR rate was 24.8%. Three SAEs related to drugs were reported.^224^ The results were promising. To explore the effects of ADXS11-001 in patients at high risk of recurrence, a phase III clinical trial was conducted, and the disease-free survival rate was focused. Although the trial was completed, the results of the trial have not been issued yet. GLBL101c, a vaccine targeting HPV-16 E7 based on heat-attenuated recombinant Lactobacillus casei, has been developed to treat patients diagnosed with CIN2. A phase IIB clinical trial was conducted to validate the effectiveness of the vaccine. Unfortunately, no significant differences were observed in the complete response rate between placebo group and GLBL101c group.^225^ Owing to the unsatisfactory result, a new vaccine, IGMKK16E7, based on GLBL101c, has been developed, which combines HPV-16-derived mutated E7Rb proteins with an anchor protein, cA, derived from *Lactococcus lactis*.^226^ A phase I/II clinical trial has shown that the complete response rate in the high-dose group is greater than that in the placebo group only in HPV-16 positive patients, but there’s no difference in the objective response rate between two groups.^227^

Viral vectors like adenoviruses (Ad), adeno-associated viruses, vaccinia viruses, and alphaviruses have been widely used in the production of vaccines because of the capability of transducing genetic information to the host. Vaccines such as MVA-E2, TA-HPV, TG4001, Vvax001, and PRGN-2009 have been validated to be effective in suppressing tumor growth clinically. Moreover, a phase II clinical trial is now recruiting patients with recurrent or metastatic CC who are pembrolizumab resistant, aiming at comparing the effectiveness of pembrolizumab with that of PRGN-2009 in combination with pembrolizumab. Ad26/35-based vaccines targeting E2, E6, and E7 of HPV-16/18 have been developed and shown great therapeutic efficacy in the TC-1 model.^228^ An Ad19a/64/5-based vaccine targeting E1, E2, E6, and E7 of HPV-16 shows superb effects in the C3 model as well.^229^ However, no Ad-based vaccines have been tested in clinical trials till now.

#### Peptide- or protein-based vaccines

Peptides and proteins can be processed by dendritic cells (DCs) to activate MHC I or MHC II, which ultimately induces CD8^+^ or CD4^+^ T-cell responses. Although vaccines based on peptides and proteins are safe and easy to manufacture, they lack high immunogenicity and may need to be combined with adjuvants to enhance immune responses. Short peptide-based vaccines are MHC-specific and need to match specific HLA, while synthetic long peptide and protein vaccines have no limitations of MHC restriction on account of the enrichment of CD8^+^ and CD4^+^ T-cell epitopes.

Peptide- or protein-based vaccines tend to be combined with other drugs in the clinic to treat hr-HPV-related cancers, especially hr-HPV-related HNSCC. ISA101 is a long peptide vaccine composed of 9 overlapping E6 peptides and 4 overlapping E7 peptides complemented by Montanide ISA-51.^230^ In a phase II trial, ISA101 together with nivolumab was tested in patients with uncurable HPV-16-positive cancer. Compared with nivolumab alone, the overall response rate (ORR) is 33%, and the median OR is 17.5 months, both of which are promising.^231^ A phase II trial is ready for recruitment to evaluate the effectiveness of ISA101 together with cemiplimab in patients with recurrent or metastatic HPV-16 positive CC. PepCan, containing 4 HPV-16 E6 synthetic peptides and adjuvant Candin, was tested in patients with HSIL.^232^ Due to the effectiveness of PepCan, a phase II trial has been conducted in patients with HSIL. The efficacy of TVGV-1 was verified in patients with HSIL in a phase IIA trial, whose results remain unknown. TVGV-1 together with the adjuvant CpG or GPI-0100 can induce tumor regression and prolong OR time in mouse models.^233^ A phase II trial is ready to be conducted to assess the efficacy and safety of TVGV-1 compared to those of the adjuvant GPI-0100 in patients with HSIL induced by HPV. PDS0101 has been tested clinically alone or in combination with pembrolizumab to evaluate its efficacy in patients with hr-HPV-associated OPSCC.

Notably, nanoparticles, which can be easily engulfed by DCs and induce immune responses, are recognized as powerful delivery platforms for peptide antigens.^234^ Polymeric nanoparticles, liposome nanoparticles and virus-like particles can be physically mixed or incorporated with antigens via encapsulation, self-assembly, absorption, and chemical conjugation. A new poly(lactide-co-glycolide)-acid nanoparticle vaccine, encapsulating HPV-16 E7_44-62_ and using ATP as an adjuvant, can inhibit tumor growth in the TC-1 model.^235^ A nanoparticle peptide vaccine composed of a pentapeptide FF-Amp-FF and its derivatives AmpFE7 (AmpFE7_49–57_ and AmpFE7_44–57_) has demonstrated to be capable of suppressing tumor growth and inducing the proliferation of T cells in the TC-1 model.^236^ Liposomes composed of 1,2-dioleoyl-3-trimethylammonium propane and HPV-16 E7_49-57_ can induce immune responses and tumor regression.^237^ An HPV-16 L1-based virus-like particle vaccine containing HPV-16 E6 and E7 epitopes can inhibit tumor growth in the TC-1 model.^238^ Interestingly, a new vaccine consisting of HPV-16 E7_44-62_ enclosed by bacterial outer membrane vesicles has shown efficacy in suppressing tumor growth and inducing antigen-specific immune responses.^239^

#### Whole-cell vaccines

DCs are considered as the most effective antigen-presenting cells in vivo and can provoke strong immune responses. DCs can also serve as adjuvants.^240^ DCs pulsed with HPV-18 E7 in vitro were subcutaneously injected into a patient with metastatic CC, which could suppress disease progression, although it couldn’t entirely cure the disease.^241^ A phase I trial has been conducted in patients with hr-HPV-positive or recurrent CC, which has shown that DC-based vaccines are promising in treating CC.^242^ DCs pulsed with HPV-16 E7 have been intended to be evaluated in patients with recurrent CC in a phase I trial. Unfortunately, the result of the trial is still unknown. DCs pulsed with HPV-16/18 E7 and keyhole limpet hemocyanin have been confirmed that humoral and cellular immunity are enhanced after immunized with the vaccine.^243^ However, difficulty in manufacturing is a limiting factor in the promotion of DC-based vaccines.

Engineered T-cell therapy can efficiently generate antigen-specific T cells in vitro and then repatriate T cells to patients to maximally induce immune responses. T-cell receptors (TCRs) can identify and target peptides derived from intracellular or extracellular proteins. The weaknesses of TCR therapy include HLA restriction and immune escape due to the deficiency of HLA elements or ingredients of antigen-processing mechanisms.^244^ Targeting E7 with TCR-T cells contributes to tumor regression in hr-HPV-related cancers.^235^ A phase II trial, which aims to evaluate the efficacy and safety of TCR gene therapy targeting HPV-16 E7 in patients diagnosed with hr-HPV-related cancers, is now recruiting participants. Coincidently, another phase I/II of TCR gene therapy targeting HPV-16 E7 in patients with CIN, tumors in situ, HPV infection, and vulvar neoplasms is recruiting participants as well.

Moreover, given that whole-tumor cell lysates can include total potential antigens, whole-tumor cell lysate-based vaccines have been developed.^245^ Heat shock protein 27 and HPV-16 E7 are transduced into TC-1 cells to develop a tumor cell lysate-based vaccine, which has shown potential for suppressing tumor growth and secreting cytokines such as IFN-γ and TNF-α.^246^

#### Nucleic acid vaccines

Nucleic acid vaccines directly introduce a DNA plasmid or mRNA sequence encoding antigens, with or without costimulatory molecules. Notably, nucleic acid vaccines induce both cellular and humoral immunity and deliver antigens in a non-MHC-restricted way.^223^

DNA-based vaccines, dependent on bacterial plasmids encoding antigens, must be delivered into the nucleus to elicit the expression of antigens and immune responses. DNA-based vaccines are featured as stable, safe, and easy to manufacture. The disadvantages of these vaccines are poor immunogenicity and inability to amplify. VGX-3100, composed of E6 and E7 proteins from HPV-16/18, has finished a phase II trial, which has shown that VGX-3100 can efficiently induce viral elimination and histopathological regression, especially in patients with CIN2/3.^247^ Moreover, high levels of IFN-γ can still be detected even 18 months after the last immunization^248^ A phase III trial has been conducted to further confirm the efficacy. About 27.6% of patients in the experimental group show complete viral elimination and histopathological regression, while the ratio in the placebo group is only 8.7%. Histologic regression is observed in 3 out of 9 patients with CIN2/3 immunized with high doses of pNGVL4a-Sig/E7(detox)/HSP70, although the result is not satisfactory.^249^ To better relieve patients from CIN2/3, the efficacy of pNGVL4a-Sig/E7(detox)/HSP70 combined with TA-HPV has been validated in the TC-1 model, which has shown that more robust immune responses are elicited with the combination of the two vaccines.^250^ Moreover, a phase I trial has been conducted to further confirm the effectiveness and safety of the combined therapy in patients with CIN3. A phase I trial is now recruiting patients with HPV-16-positive ISIL or tumors in situ to validate the efficacy of different doses of pNGVL4aCRTE6E7L2 with or without TA-CIN. After chemoradiation, patients with CC receive the immunization with INO-3112, which results in humoral and cellular responses targeting HPV and the elimination of HPV DNA.^251^ Besides, the efficacy of INO-3112 combined with durvalumab has been proven in patients with recurrent or metastatic HPV-associated cancers. The ORR is 21%. However, unfortunately, the study is interrupted due to the low ORR in patients with CC.^252^ GX188E, proven to be effective in treating patients with HPV-16/18-associated cancers, has been combined with pembrolizumab to treat patients suffering from HPV-16 or 18 positive advanced CC. The ORR is 42%, and the combined therapy is relatively safe for patients.^253,254^

RNA-based vaccines are based on RNA sequences of the antigen. When taken up by antigen-presenting cells and other target cells, vaccines induce expression of antigenic proteins. Owing to the instability and inability to spread intercellularly, RNA-based vaccines are always packaged into vectors, including cationic lipids and polymer materials. HPV RNA-lipoplex (RNA-LPX) can induce robust T-cell responses via intravenous injection and can persistently suppress tumor growth in the TC-1 model.^255^ Unmodified or nucleoside-modified non-replicating mRNA as well as self-amplifying mRNA vaccines, which encode HPV-16 E7 and herpes simplex virus type 1 glycoprotein D, are encapsulated by lipid nanoparticles. The vaccine has shown that it can suppress tumor growth, provide prolonged antitumor protection, and elicit strong immune responses in both subcutaneous and orthotopic tumor models.^256^ mHTV, an mRNA vaccine composed of E6 and E7 proteins from HPV-16/18, has displayed the ability to suppress tumor growth, modulate the tumor microenvironment, and offer continuous protection in subcutaneous and orthotopic tumor models in mice, especially in those that receive immunization via subcutaneous injection. Moreover, the vaccine can provoke antigen-specific immune responses in rhesus monkeys.^257^ The mRNA-LNP vaccine, which encodes HPV-16 E7, can induce robust HPV-specific CD8^+^ T-cell responses and IFNγ secretion in an hr-HPV-related OPSCC mouse model. In addition, mRNA-LNP vaccine combined with immune checkpoint blockade can better promote tumor regression.^258^

### Significance of hr-HPV in prognosis

According to a meta-analysis, hr-HPV positivity is associated with a lower cancer stage, squamous cell carcinoma histology, and better disease-free survival and OR rate. Nevertheless, it has no connection with the reoccurrence of CC.^259^ Moreover, compared with HPV-16 positivity, HPV-18 positivity is associated with a poor prognosis, especially for early-stage cancers.^260^ hr-HPV positivity indicating better prognosis of cancers can be applied to clinic in the future. Research has confirmed that hr-HPV-positive patients have a better OR rate than hr-HPV-negative patients in OPSCC.^261^ Since hr-HPV contributes to the epigenetic reprogramming of host cells, hr-HPV-related DNA methylation has also been applied to clinic. hr-HPV-related DNA methylation is explored to predict the prognosis of patients diagnosed with CC via gene set enrichment analysis, Cox regression analysis, and Kaplan–Meier survival analysis.^262^ Utilizing E7 sequences, a marker for circulating hr-HPV DNA, to predict the prognosis of patients diagnosed with CC is a new method identified in a prospective cohort of CC. In this study, researchers have found that remaining circulating hr-HPV DNA after treatment is closely related to poor prognosis of patients suffering from CC and can predict the reoccurrence of CC.^263^

Owing to the widespread use of hr-HPV screening and prophylactic vaccines, the occurrence of hr-HPV-related cancers, especially CC, has declined significantly. Unlike CC, the detection of hr-HPV in HNSCC depends on the original sites of tumors. hr-HPV has become a potent biomarker in OPSCC, which indicates a favorable prognosis and assists in staging of cancers. Nowadays, detection of HPV can adopt p16 immunohistochemistry, PCR, RNA in situ hybridization, and single-versus multimodality HPV analysis.^264^ Circulating hr-HPV DNA is correlated with tumor burden and prognosis. Low levels of hr-HPV DNA imply low hr-HPV copy numbers and hr-HPV integration.^265^ Therefore, circulating hr-HPV DNA may be a new method for detecting hr-HPV-related cancers in the future.

## Conclusion and perspective

In this review, we summarize the detailed oncogenic signaling pathways of hr-HPV and its indispensable role in clinic, including screening, diagnosis, prevention, and treatment of cancers. hr-HPV infection results in the occurrence of cancers mainly dependent on early proteins, among which E6 and E7 are recognized as main oncoproteins in the process of oncogenesis. They can disturb the cell cycle, affect cell proliferation, inhibit apoptosis, and alter cellular metabolism, leading to the occurrence and development of cancers.^75^

Several types of vaccines have been developed and popularized to prevent hr-HPV-related cancers, from which large quantities of people have benefited. Given the popularization of prophylactic vaccines, the incidence of hr-HPV-related cancers has declined dramatically. A majority of therapeutic vaccines targeting E6 and E7 proteins have been developed. Moreover, vaccines tend to be utilized in combination with other therapies such as radiotherapy, chemotherapy, and immunotherapy. To exert the antitumor effects of vaccines to the largest extent, an increasing number of clinical trials have been conducted to validate the safety and efficacy of combined therapy for treating hr-HPV-related cancers.

Apart from vaccines targeting hr-HPVs, other therapies have been applied clinically as well (Fig. 4). Immunotherapy, such as immune checkpoint blockade therapy and adoptive T-cell therapy, aiming to reverse the immunosuppressive environment, have been widely studied. Immune checkpoint blockades, such as nivolumab, pembrolizumab, tremelimumab, and durvalumab, have been tested in clinic alone or in combination with standard therapies in hr-HPV-related cancers to verify their efficacy. Research has proved that inhibition of CD96 can enhance the blockade of PD-1 and boost the function of CD8^+^ T cells, which provides inspiration for therapy.^266^ Adoptive cell therapy, including tumor-infiltrating lymphocytes, genetically modified TCRs, and T cells with chimeric antigen receptors, also offers a prospective therapy for hr-HPV-related cancers. Although numerous clinical trials have been conducted to verify the efficacy and safety of these therapies,^267^ their clinical application remains controversial. In addition to immunotherapy, various drugs have been applied as well. Bevacizumab, which belongs to anti-VEGF antibodies, has been widely applied, particularly to patients diagnosed with recurrent or metastatic cancers. REBACIN has shown its great potential in eliminating HPV infection via suppressing E6 and E7 genes.^268^ Nocardia rubra cell wall skeleton, recognized as National Category II New Drug in China, can impair T-cell exhaustion and enhance local immune responses in patients undergoing HPV infection or diagnosed with CIN.^269^ Imiquimod, considered as an agonist of TLR7, can activate DCs and promote the secretion of IFNγ and TNFα.^270^ A phase II clinical trial has witnessed its efficacy in eliminating HPV infection and treating HSIL, especially in combination with 9-valent HPV vaccines.^271^ Gene silencing and editing, including meganucleases, transcription activator-like effector nucleases, zinc finger nucleases, and clustered regularly interspaced short palindromic repeat (CRISPR)-associated Cas9 has provided new insight into treating hr-HPV-related cancers. Research has found that shRNA or siRNA targeting E6 and/or E7 dramatically downregulates the expression of E6 and/or E7 and upregulates the expression of p53 and pRb simultaneously in hr-HPV-positive HNSCC cells. CRISPR-Cas9 targeting E7 delivered by nanoparticles can interfere with viral genome constantly and demonstrate its efficacy in eliminating tumor in a CC xenograft mouse model.^272^ Though CRISPR-Cas9 shows potential in treating hr-HPV-related cancers, it is limited by low efficiency of editing. Enhancing the gene editing efficiency and specificity needs to be considered in the future.^273^ Physical therapies such as radiotherapy and hyperthermia have been noticed. A clinical trial has been conducted to compare the efficacy of radiotherapy with that of hyperthermia plus radiotherapy. Hyperthermia plus radiotherapy has the better local control efficacy in locally advanced pelvic tumors, especially in CC.^274^ In addition, hyperthermia downregulates E6 and contributes to the accumulation of p53, triggering the occurrence of p53-mediated apoptosis and G2-phase arrest in HPV-positive cells, which may benefit patients and decrease the normal tissue damage maximally.^275^ Research has found that the stability of IGFBP1 can be destroyed and the expression of E7 can thus be downregulated by heat treatment, which provides a new strategy to treat hr-HPV-related cancers on the basis of heat treatment.^276^

Considering that a high proportion of cancers, such as CC and HNSCC, are caused by persistent infection with hr-HPVs and that a large number of people suffer from cancers, deeper research on hr-HPV is of necessity. Despite the fact that various therapeutic vaccines are produced, none of them have been widely used due to the safety and adverse events. Enhancing the potency and safety of vaccines to benefit people suffering from hr-HPV-related cancers may be the focus of future studies.
